# 2-Hy­droxy-2-methyl-1-phenyl­indolin-3-one

**DOI:** 10.1107/S1600536811045302

**Published:** 2011-11-09

**Authors:** Andrej Pevec, Stanislav Kafka, Janez Košmrlj

**Affiliations:** a Faculty of Chemistry and Chemical Technology, University of Ljubljana, SI-1000 Ljubljana, Slovenia; b Department of Chemistry, Faculty of Technology, Tomas Bata University in Zlin, Zlin 76272, Czech Republic

## Abstract

In the title compound, C_15_H_13_NO_2_, the indole and benzene rings make a dihedral angle of 60.61 (4)°. In the crystal, dimeric pairs (twofold symmetry) are formed *via* O—H⋯O hydrogen bonds.

## Related literature

For naturally occurring 2-hy­droxy­indol-3-ones, see: Bhakuni *et al.* (1991 ▶). For inter­mediates of the 2-hy­droxy­indol-3-one substructure in the total syntheses of some natural products including (+)-isatisine A, (±)-mersicarpine, hinckdentine A, mitomycin and others, see: Karadeolian & Kerr (2010 ▶); Magolan *et al.* (2008 ▶); Higuchi *et al.* (2009 ▶); Colandrea *et al.* (2003 ▶); Kawasaki *et al.* (2004 ▶). For recent syntheses of 2-hy­droxy­indol-3-ones, see: Coldham *et al.* (2010 ▶); Higuchi *et al.* (2010 ▶); Cariou *et al.* (2007 ▶); Hewitt & Shao (2006 ▶); Altinis Kiraz *et al.* (2004 ▶). For the synthesis of the title compound, see: Kafka *et al.* (2001 ▶).
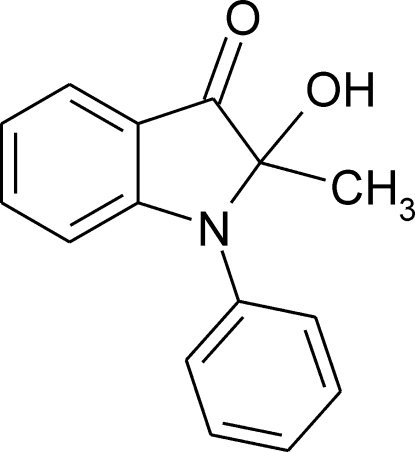


## Experimental

### 

#### Crystal data

C_15_H_13_NO_2_
*M* *_r_* = 239.26
Orthorhombic, 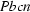

*a* = 17.0146 (4) Å
*b* = 9.2193 (2) Å
*c* = 15.3843 (4) Å
*V* = 2413.22 (10) Å^3^
*Z* = 8
Mo *K*α radiation
μ = 0.09 mm^−1^
*T* = 295 K
0.55 × 0.40 × 0.30 mm

#### Data collection

Nonius KappaCCD area-detector diffractometer
Absorption correction: multi-scan (*SCALEPACK*; Otwinowski & Minor, 1997 ▶) *T*_min_ = 0.953, *T*_max_ = 0.974
5144 measured reflections
2745 independent reflections
1890 reflections with *I* > 2σ(*I*)
*R*_int_ = 0.022

#### Refinement

*R*[*F* ^2^ > 2σ(*F* ^2^)] = 0.043
*wR*(*F* ^2^) = 0.116
*S* = 1.04
2745 reflections
165 parameters
H-atom parameters constrained
Δρ_max_ = 0.15 e Å^−3^
Δρ_min_ = −0.16 e Å^−3^

### 

Data collection: *COLLECT* (Hooft, 1998 ▶); cell refinement: *DENZO* and *SCALEPACK* (Otwinowski & Minor, 1997 ▶); data reduction: *DENZO* and *SCALEPACK*; program(s) used to solve structure: *SIR92* (Altomare *et al.*, 1999 ▶); program(s) used to refine structure: *SHELXL97* (Sheldrick, 2008 ▶); molecular graphics: *PLATON* (Spek, 2009 ▶) and *DIAMOND* (Brandenburg, 1999 ▶); software used to prepare material for publication: *WinGX* (Farrugia, 1999 ▶).

## Supplementary Material

Crystal structure: contains datablock(s) I, global. DOI: 10.1107/S1600536811045302/tk5007sup1.cif

Structure factors: contains datablock(s) I. DOI: 10.1107/S1600536811045302/tk5007Isup2.hkl

Supplementary material file. DOI: 10.1107/S1600536811045302/tk5007Isup3.cml

Additional supplementary materials:  crystallographic information; 3D view; checkCIF report

## Figures and Tables

**Table 1:** Hydrogen-bond geometry (Å, °)

*D*—H⋯*A*	*D*—H	H⋯*A*	*D*⋯*A*	*D*—H⋯*A*
O2—H2⋯O1^i^	0.82	2.12	2.9014 (15)	159

Symmetry code: (i) 
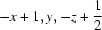
.

**Fig. 1.**
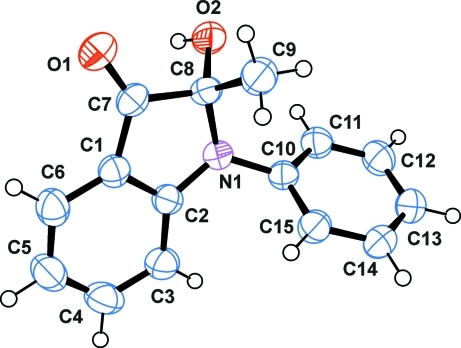
A view of (I) showing the atom-numbering scheme. Displacement ellipsoids are drawn at the 50% probability level.

**Fig. 2.**
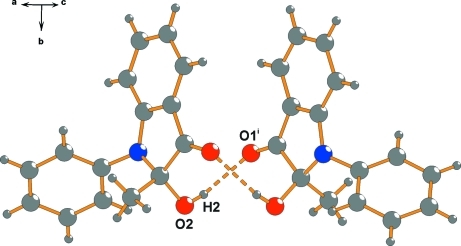
Association betweem two molecules of (I) via O—H···O hydrogen bonds denoted by dashed lines. Symmetry code: (i) -x + 1, y, -z + 1/2.

## supplementary crystallographic information

### Comment

The title compound, (I) (Fig. 1), was prepared as a part of a project focusing on molecular rearrangements of 3-hydroxyquinoline-2,4(1*H*,3*H*)-diones by the action of aqueous potassium hydroxide on 3-hydroxy-3-methyl-1-phenylquinoline-2,4(1*H*,3*H*)-dione in the presence of air (Kafka *et al.*, 2001). Compounds containing the 2-hydroxyindol-3-one substructure are important intermediates in the total syntheses of some natural products including (+)-isatisine A, (±)-mersicarpine, hinckdentine A, mitomycin and others (Karadeolian & Kerr, 2010; Magolan *et al.*, 2008; Higuchi *et al.*, 2009; Colandrea *et al.*, 2003; Kawasaki *et al.*, 2004). New synthetic approaches towards 2-hydroxyindol-3-ones have been reported recently (Coldham *et al.*, 2010; Higuchi *et al.*, 2010; Cariou *et al.*, 2007; Hewitt & Shao, 2006; Altinis Kiraz *et al.*, 2004). A related 2-hydroxyindol-3-one compound, alkaloid melochicorine, was found in the plant *Melochicoria corchorifolia* (Bhakuni *et al.*, 1991)

In the crystal structure of (I) two molecules are connected by two intermolecular O—H···O hydrogen bonds (Fig. 2 & Table 1). The indole and benzene units make a dihedral angle of 60.61 (4)°. The aromatic rings have normal hydrophobic contacts with each other without any stacking interactions.

### Experimental

A mixture of 3-hydroxy-3-methyl-1-phenylquinoline-2,4(1*H*,3*H*)-dione (1.34 g, 5.0 mmol) in 1.3 *M* aqueous potassium hydroxide (30 ml) and benzene (120 ml) was vigorously stirred in the presence of air at room temperature for 30 min. Layers were separated, and the aqueous phase was extracted with benzene (5 *x* 20 ml). The combined organic layer was dried over K_2_CO_3_. The solvent was evaporated to dryness and the residue was crystallized from a mixture of benzene and cyclohexane to give crystals of the title compound (0.62 g, 2.6 mmol, 52%).

### Refinement

All H atoms were positioned geometrically and refined using a riding model, with C—H bond lengths constrained to 0.93 (aromatic-C—H) or 0.96 Å (methyl-C—H), and O—H = 0.82 Å, and with *U*_iso_(H) values of 1.2*U*_eq_(C) [for aromatic-H] or 1.5*U*_eq_(C) [for OH and methyl-H].

### Crystal data

**Table:**

C_15_H_13_NO_2_	*D*_x_ = 1.317 Mg m^−^^3^
*M**_r_* = 239.26	Melting point = 397–399 K
Orthorhombic, *P**b**c**n*	Mo *K*α radiation, λ = 0.71073 Å
Hall symbol: -P 2n 2ab	Cell parameters from 3116 reflections
*a* = 17.0146 (4) Å	θ = 1.0–27.5°
*b* = 9.2193 (2) Å	µ = 0.09 mm^−^^1^
*c* = 15.3843 (4) Å	*T* = 295 K
*V* = 2413.22 (10) Å^3^	Prism, green
*Z* = 8	0.55 × 0.40 × 0.30 mm
*F*(000) = 1008	

### Data collection

**Table:**

Nonius KappaCCD area-detector diffractometer	2745 independent reflections
Radiation source: fine-focus sealed tube	1890 reflections with *I* > 2σ(*I*)
graphite	*R*_int_ = 0.022
φ and ω scans	θ_max_ = 27.5°, θ_min_ = 3.5°
Absorption correction: multi-scan (*SCALEPACK*; Otwinowski & Minor, 1997)	*h* = −22→21
*T*_min_ = 0.953, *T*_max_ = 0.974	*k* = −11→11
5144 measured reflections	*l* = −19→19

### Refinement

**Table:**

Refinement on *F*^2^	Primary atom site location: structure-invariant direct methods
Least-squares matrix: full	Secondary atom site location: difference Fourier map
*R*[*F*^2^ > 2σ(*F*^2^)] = 0.043	Hydrogen site location: inferred from neighbouring sites
*wR*(*F*^2^) = 0.116	H-atom parameters constrained
*S* = 1.04	*w* = 1/[σ^2^(*F*_o_^2^) + (0.0545*P*)^2^ + 0.3729*P*] where *P* = (*F*_o_^2^ + 2*F*_c_^2^)/3
2745 reflections	(Δ/σ)_max_ = 0.0001
165 parameters	Δρ_max_ = 0.15 e Å^−^^3^
0 restraints	Δρ_min_ = −0.16 e Å^−^^3^

### Special details

**Table:**

Experimental. 210 frames in 4 sets of φ scans + ω scans. Rotation/frame = 2 °. Crystal-detector distance = 31 mm. Measuring time = 20 s/°.
Geometry. All s.u.'s (except the s.u. in the dihedral angle between two l.s. planes) are estimated using the full covariance matrix. The cell s.u.'s are taken into account individually in the estimation of s.u.'s in distances, angles and torsion angles; correlations between s.u.'s in cell parameters are only used when they are defined by crystal symmetry. An approximate (isotropic) treatment of cell s.u.'s is used for estimating s.u.'s involving l.s. planes.
Refinement. Refinement of *F*^2^ against ALL reflections. The weighted *R*-factor *wR* and goodness of fit *S* are based on *F*^2^, conventional *R*-factors *R* are based on *F*, with *F* set to zero for negative *F*^2^. The threshold expression of *F*^2^ > 2σ(*F*^2^) is used only for calculating *R*-factors(gt) *etc*. and is not relevant to the choice of reflections for refinement. *R*-factors based on *F*^2^ are statistically about twice as large as those based on *F*, and *R*- factors based on ALL data will be even larger.

### Fractional atomic coordinates and isotropic or equivalent isotropic displacement parameters (Å^2^)

**Table:**

	*x*	*y*	*z*	*U*_iso_*/*U*_eq_	
O1	0.58436 (6)	0.68168 (12)	0.29672 (6)	0.0524 (3)	
O2	0.54290 (7)	0.87398 (11)	0.15350 (7)	0.0562 (3)	
H2	0.5002	0.8334	0.1584	0.084*	
N1	0.59892 (7)	0.68372 (12)	0.06732 (7)	0.0406 (3)	
C1	0.59694 (8)	0.51234 (15)	0.17738 (9)	0.0416 (3)	
C2	0.60064 (8)	0.53713 (15)	0.08779 (9)	0.0404 (3)	
C3	0.60139 (9)	0.42005 (16)	0.03007 (11)	0.0524 (4)	
H3	0.6043	0.4339	−0.0297	0.063*	
C4	0.59754 (10)	0.28280 (17)	0.06584 (12)	0.0614 (5)	
H4	0.5981	0.2033	0.0286	0.074*	
C5	0.59282 (10)	0.25788 (18)	0.15492 (13)	0.0620 (5)	
H5	0.5900	0.1635	0.1760	0.074*	
C6	0.59237 (9)	0.37246 (16)	0.21147 (11)	0.0526 (4)	
H6	0.5891	0.3574	0.2711	0.063*	
C7	0.59389 (8)	0.65226 (15)	0.22002 (9)	0.0405 (3)	
C8	0.60272 (8)	0.76939 (15)	0.14873 (9)	0.0407 (3)	
C9	0.68031 (10)	0.84809 (18)	0.15897 (11)	0.0580 (4)	
H9A	0.6833	0.8899	0.2160	0.087*	
H9B	0.6840	0.9235	0.1161	0.087*	
H9C	0.7228	0.7808	0.1512	0.087*	
C10	0.63405 (8)	0.74129 (14)	−0.00952 (8)	0.0392 (3)	
C11	0.59874 (9)	0.85663 (15)	−0.05174 (10)	0.0472 (4)	
H11	0.5505	0.8915	−0.0326	0.057*	
C12	0.63529 (10)	0.92009 (17)	−0.12243 (11)	0.0561 (4)	
H12	0.6115	0.9979	−0.1505	0.067*	
C13	0.70639 (10)	0.86900 (16)	−0.15140 (10)	0.0534 (4)	
H13	0.7310	0.9128	−0.1986	0.064*	
C14	0.74101 (9)	0.75336 (16)	−0.11069 (10)	0.0519 (4)	
H14	0.7889	0.7181	−0.1307	0.062*	
C15	0.70513 (9)	0.68883 (16)	−0.03994 (9)	0.0473 (4)	
H15	0.7288	0.6100	−0.0127	0.057*	

### Atomic displacement parameters (Å^2^)

**Table:**

	*U*^11^	*U*^22^	*U*^33^	*U*^12^	*U*^13^	*U*^23^
O1	0.0523 (6)	0.0653 (7)	0.0397 (6)	0.0011 (5)	0.0016 (4)	−0.0021 (5)
O2	0.0597 (7)	0.0457 (6)	0.0633 (7)	0.0129 (5)	0.0195 (6)	0.0033 (5)
N1	0.0443 (6)	0.0384 (6)	0.0389 (6)	−0.0010 (5)	0.0037 (5)	−0.0020 (5)
C1	0.0358 (7)	0.0431 (8)	0.0459 (8)	0.0021 (6)	0.0026 (6)	0.0025 (6)
C2	0.0351 (7)	0.0387 (7)	0.0475 (8)	−0.0014 (6)	0.0029 (6)	−0.0022 (6)
C3	0.0548 (9)	0.0483 (9)	0.0542 (9)	−0.0052 (7)	0.0057 (7)	−0.0088 (7)
C4	0.0595 (10)	0.0419 (9)	0.0829 (13)	−0.0044 (7)	0.0082 (9)	−0.0142 (8)
C5	0.0594 (10)	0.0403 (8)	0.0862 (13)	0.0010 (7)	0.0092 (9)	0.0071 (8)
C6	0.0465 (8)	0.0496 (9)	0.0618 (9)	0.0053 (7)	0.0063 (7)	0.0127 (8)
C7	0.0314 (7)	0.0499 (8)	0.0401 (7)	0.0013 (6)	0.0018 (6)	−0.0006 (6)
C8	0.0405 (7)	0.0397 (7)	0.0419 (7)	0.0012 (6)	0.0065 (6)	−0.0058 (6)
C9	0.0580 (10)	0.0628 (10)	0.0532 (9)	−0.0181 (8)	0.0073 (7)	−0.0130 (8)
C10	0.0424 (7)	0.0395 (7)	0.0357 (7)	−0.0021 (6)	0.0003 (6)	−0.0035 (6)
C11	0.0444 (8)	0.0436 (8)	0.0536 (9)	0.0038 (6)	0.0024 (7)	0.0017 (7)
C12	0.0632 (10)	0.0459 (9)	0.0592 (10)	0.0057 (8)	0.0005 (8)	0.0119 (7)
C13	0.0637 (10)	0.0485 (9)	0.0479 (9)	−0.0026 (7)	0.0105 (7)	0.0064 (7)
C14	0.0497 (9)	0.0532 (9)	0.0530 (9)	0.0035 (7)	0.0121 (7)	0.0001 (7)
C15	0.0485 (8)	0.0476 (8)	0.0457 (8)	0.0077 (7)	0.0031 (6)	0.0058 (6)

### Geometric parameters (Å, °)

**Table:**

O1—C7	1.2216 (16)	C7—C8	1.5464 (19)
O2—C8	1.4040 (16)	C8—C9	1.515 (2)
O2—H2	0.8200	C9—H9A	0.9600
N1—C2	1.3880 (17)	C9—H9B	0.9600
N1—C10	1.4271 (17)	C9—H9C	0.9600
N1—C8	1.4821 (17)	C10—C11	1.3833 (19)
C1—C6	1.3943 (19)	C10—C15	1.3840 (19)
C1—C2	1.398 (2)	C11—C12	1.383 (2)
C1—C7	1.4481 (19)	C11—H11	0.9300
C2—C3	1.398 (2)	C12—C13	1.373 (2)
C3—C4	1.381 (2)	C12—H12	0.9300
C3—H3	0.9300	C13—C14	1.370 (2)
C4—C5	1.392 (3)	C13—H13	0.9300
C4—H4	0.9300	C14—C15	1.383 (2)
C5—C6	1.368 (2)	C14—H14	0.9300
C5—H5	0.9300	C15—H15	0.9300
C6—H6	0.9300		
			
C8—O2—H2	109.5	O2—C8—C7	111.84 (11)
C2—N1—C10	122.77 (11)	N1—C8—C7	102.88 (10)
C2—N1—C8	109.04 (11)	C9—C8—C7	110.21 (12)
C10—N1—C8	118.92 (10)	C8—C9—H9A	109.5
C6—C1—C2	121.62 (13)	C8—C9—H9B	109.5
C6—C1—C7	130.66 (14)	H9A—C9—H9B	109.5
C2—C1—C7	107.61 (12)	C8—C9—H9C	109.5
N1—C2—C3	127.44 (14)	H9A—C9—H9C	109.5
N1—C2—C1	112.44 (12)	H9B—C9—H9C	109.5
C3—C2—C1	120.02 (13)	C11—C10—C15	119.30 (13)
C4—C3—C2	116.99 (15)	C11—C10—N1	119.54 (12)
C4—C3—H3	121.5	C15—C10—N1	121.07 (12)
C2—C3—H3	121.5	C12—C11—C10	119.95 (14)
C3—C4—C5	123.10 (16)	C12—C11—H11	120.0
C3—C4—H4	118.4	C10—C11—H11	120.0
C5—C4—H4	118.4	C13—C12—C11	120.44 (14)
C6—C5—C4	119.92 (15)	C13—C12—H12	119.8
C6—C5—H5	120.0	C11—C12—H12	119.8
C4—C5—H5	120.0	C14—C13—C12	119.84 (14)
C5—C6—C1	118.33 (15)	C14—C13—H13	120.1
C5—C6—H6	120.8	C12—C13—H13	120.1
C1—C6—H6	120.8	C13—C14—C15	120.34 (14)
O1—C7—C1	129.80 (13)	C13—C14—H14	119.8
O1—C7—C8	122.88 (13)	C15—C14—H14	119.8
C1—C7—C8	107.30 (11)	C14—C15—C10	120.11 (14)
O2—C8—N1	112.24 (11)	C14—C15—H15	119.9
O2—C8—C9	107.31 (12)	C10—C15—H15	119.9
N1—C8—C9	112.40 (11)		
			
C10—N1—C2—C3	−31.0 (2)	C10—N1—C8—C9	−37.57 (17)
C8—N1—C2—C3	−177.20 (14)	C2—N1—C8—C7	−8.44 (13)
C10—N1—C2—C1	152.51 (12)	C10—N1—C8—C7	−156.10 (11)
C8—N1—C2—C1	6.35 (15)	O1—C7—C8—O2	−50.00 (18)
C6—C1—C2—N1	175.46 (12)	C1—C7—C8—O2	128.43 (12)
C7—C1—C2—N1	−1.03 (15)	O1—C7—C8—N1	−170.66 (12)
C6—C1—C2—C3	−1.3 (2)	C1—C7—C8—N1	7.77 (13)
C7—C1—C2—C3	−177.77 (13)	O1—C7—C8—C9	69.29 (16)
N1—C2—C3—C4	−175.56 (13)	C1—C7—C8—C9	−112.29 (12)
C1—C2—C3—C4	0.6 (2)	C2—N1—C10—C11	144.73 (13)
C2—C3—C4—C5	0.2 (2)	C8—N1—C10—C11	−72.24 (17)
C3—C4—C5—C6	−0.4 (3)	C2—N1—C10—C15	−38.80 (19)
C4—C5—C6—C1	−0.2 (2)	C8—N1—C10—C15	104.23 (15)
C2—C1—C6—C5	1.0 (2)	C15—C10—C11—C12	−1.3 (2)
C7—C1—C6—C5	176.62 (14)	N1—C10—C11—C12	175.20 (13)
C6—C1—C7—O1	−2.2 (3)	C10—C11—C12—C13	0.3 (2)
C2—C1—C7—O1	173.84 (14)	C11—C12—C13—C14	0.7 (3)
C6—C1—C7—C8	179.50 (14)	C12—C13—C14—C15	−0.7 (2)
C2—C1—C7—C8	−4.44 (14)	C13—C14—C15—C10	−0.3 (2)
C2—N1—C8—O2	−128.83 (11)	C11—C10—C15—C14	1.3 (2)
C10—N1—C8—O2	83.51 (14)	N1—C10—C15—C14	−175.13 (13)
C2—N1—C8—C9	110.09 (13)		

### Hydrogen-bond geometry (Å, °)

**Table:**

*D*—H···*A*	*D*—H	H···*A*	*D*···*A*	*D*—H···*A*
O2—H2···O1^i^	0.82	2.12	2.9014 (15)	159

Symmetry codes: (i) −*x*+1, *y*, −*z*+1/2.
